# Comprehensive assessment gene signatures for clear cell renal cell carcinoma prognosis

**DOI:** 10.1097/MD.0000000000012679

**Published:** 2018-11-02

**Authors:** Peng Chang, Zhitong Bing, Jinhui Tian, Jingyun Zhang, Xiuxia Li, Long Ge, Juan Ling, Kehu Yang, Yumin Li

**Affiliations:** a School of Life Sciences, Lanzhou University; b Lanzhou University Second Hospital; c Evidence Based Medicine Center, School of Basic Medical Science of Lanzhou University; d Key Laboratory of Evidence Based Medicine and Knowledge Translation of Gansu Province; e School of Public Health, Lanzhou University, Lanzhou, China.

**Keywords:** clear cell renal cell carcinoma, Cox regression, gene regulatory network, least absolute shrinkage and selection operator, prognosis

## Abstract

There are many prognostic gene signature models in clear cell renal cell carcinoma (ccRCC). However, different results from various methods and samples are hard to contribute to clinical practice. It is necessary to develop a robust gene signature for improving clinical practice in ccRCC.

A method was proposed to integrate least absolute shrinkage and selection operator and multiple Cox regression to obtain mRNA and microRNA signature from the cancer genomic atlas database for predicting prognosis of ccRCC. The gene signature model consisted by 5 mRNAs and 1 microRNA was identified. Prognosis index (PI) model was constructed from RNA expression and median value of PI is used to classified patients into high- and low-risk groups.

The results showed that high-risk patients showed significantly decrease survival comparison with low-risk groups [hazard ratio (HR) =7.13, 95% confidence interval = 3.71–13.70, *P* < .001]. As the gene signature was mainly consisted by mRNA, the validation data can use transcriptomic data to verify. For comparison of the performance with previous works, other gene signature models and 4 datasets of ccRCC were retrieved from publications and public database. For estimating PI in each model, 3 indicators including HR, concordance index , and the area under the curve of receiver operating characteristic for 3 years were calculated across 4 independent datasets.

The comparison results showed that the integrative model from our study was more robust than other models via comprehensive analysis. These findings provide some genes for further study their functions and mechanisms in ccRCC tumorigenesis and malignance, and may be useful for effective clinical decision making of ccRCC patients.

## 1 Introduction

Renal cell carcinoma (RCC) is a frequent malignant tumor of the adult kidney. According to cancer statistics in 2018, 65,340 new kidney cancer patients and 14,970 deaths.^[1]^ Comparison to 10 years ago (2007), 51,190 individuals suffered from kidney cancer and 12,890 died.^[2]^ In 10 years, the morbidity and mortality of patients with renal cancer have not been significantly changed. One of the important reasons is that RCC is a highly heterogeneous set of disease. Of these subtypes of RCCs, The clear-cell renal cell carcinoma (ccRCC) is one of most common subtypes, accounting for approximately 70% to 80% of the whole RCC.^[3]^ Thus, identification of robust biomarkers for ccRCC prognosis is necessary.

In an age of precision medicine, molecular subtype and gene signature can provide new insight for clinical strategy and drug development. High-throughput gene sequencing technology provides us with a powerful tool to find genetic differences among different patients. Therefore, different strategies can be used to treat the patient in molecular level. Nevertheless, different approaches using by different groups have produced many different prognostic biomarkers for ccRCC.^[3–24]^ How do we decide which gene signature is effective against the current ccRCC, which gene signature is more universal? In this study, a least absolute shrinkage and selection operator (LASSO) penalized Cox regression analysis method was combined with multivariate Cox regression to obtain a set of gene biomarkers and compared it with other gene signature from publications. In addition, there are many studies in microRNA prognostic signature.^[15,25–28]^ Thus, we also integrated microRNA and mRNA expression for predicting prognosis of ccRCC.

In this study, we select data from 2 common databases of the cancer genome atlas (TCGA) and gene expression omnibus (GEO) database as training data sets and validating datasets, respectively. Other 3 gene signatures were also tested with these 2 database sets. To exclude methodological heterogeneity, we selected 3 studies of gene prognostic biomarkers that were obtained using Cox regression. In the 3 studies, Yao et al^[9]^ find 3 genes for prognosis of ccRCC, Boguslawska et al^[22]^ find 10 genes and Zhan et al^[21]^ find 5 genes. In our study, we found 5 mRNAs and 1 microRNA can predict prognosis of ccRCC. Interestingly, each of the gene signature has no intersection, but they performed well in validating the data set.

Here we demonstrate that the gene signature from LASSO combined multivariate Cox regression has more stability and universal in prognosis of ccRCC. These may be helpful for selecting high-risk ccRCC patients for better clinical decision making and provide useful biomarkers for downstream experimental.

## 2 Methods

### 2.1 Data collected and preprocessing

The microRNA expression proliferation and mRNA expression proliferation are collected TCGA database (https://portal.gdc.cancer.gov/). The mRNA dataset is performed by the Illumina Hiseq platform. And microRNA dataset is also performed by Illumina Hiseq platform. In ccRCC cohort of TCGA database, RNASeqV2 expression data contain 20,530 genes and microRNA expression data contain 1046 microRNAs. The patients without survival time and event information were excluded. The samples with mRNA expression contained 510 patients with primary tumor and 70 patients with solid tissue normal. The samples with microRNA expression contained 254 patients with primary tumor and 71 patients with solid tissue normal. We used both mRNA and miRNA samples as training dataset (n = 239). Validation data are collected from GEO database. GSE22541 contains 68 samples which include 24 primary and 44 metastasis samples.^[29]^ And this dataset employed Affymetrix Human Genome U133 Plus 2.0 Array. This work did not directly use tissues from patients or animals.

### 2.2 Gene differential expression analysis

After differential gene expression analysis, there are 4205 mRNAs and 59 microRNAs with differential expression comparison of normal tissue. In this study, we selected differential expression genes with fold-change >1.5 and false discovery rate (FDR) <0.01 as candidate genes for next step. The microRNA and mRNA differential expression are assayed by R package by “limma”^[30]^ from Bioconductor 2.14.

### 2.3 Univariate Cox regression gene test

Firstly, univariate Cox regression and survival analysis are applied to analyze clinical factors and each differential expression gene. For clinical factors, we employed univariate Cox regression to test hazard ratio (HR). And survival analysis using Kaplan-Meier is applied to analyze clinical factor significant difference by log-rank (2-sided test). For estimating the clinical factor in ccRCC patients, HR >1 is considered as risk increasing group and HR <1 is considered as risk-decreasing factor.

### 2.4 Multivariate Cox regression for clinical factor and LASSO Cox regression for RNAs

For univariate Cox regression clinical factor, we screened the factor with Wald test *P* < .05 as candidate for multivariate Cox regression.

For mRNA and miRNA expression, we filtered the RNA with Wald test *P* < .05 as candidate for LASSO Cox regression. By univariate Cox regression filtering, 2498 mRNAs and 18 miRNAs were selected to submit to LASSO. LASSO is performed by R package of “glmnet.” LASSO is employed to filter 2498 mRNAs and 18 miRNAs, respectively. After 10,000 iterations and 10 folds cross-validation, 16 mRNAs and 9 miRNAs are obtained.

### 2.5 Integrative mRNAs and miRNAs for predicting survival of ccRCC

For investigating integrative model of miRNAs and mRNAs, ccRCC patients who both include mRNA and miRNA expression are selected. There are 239 samples including for assay the integrative model. We employed multivariate Cox regression for 25 RNAs (16 mRNAs and 9 microRNAs), and we obtained 5 mRNAs and 1 microRNA-independent predictors for ccRCC.

### 2.6 Literature reviews

Prognostic model search was employed by PubMed. The following terms were searched in PubMed: ({“clear cell renal cell carcinoma” OR “clear cell renal cell cancer” OR “clear cell renal cell tumor” OR “clear cell renal cell tumour” OR “clear cell kidney cell carcinoma” OR “clear cell kidney cell cancer” OR “clear cell kidney cell tumor” OR “clear cell renal cell tumour”} AND {“gene expression” OR “gene signature” OR “gene proliferation” OR “microarray” OR “high-throughput” OR “microRNA expression” OR “mRNA expression”} AND{“survival” OR “survivor” OR “outcome” OR “prognosis” OR “prognostic” OR “prediction”} AND {“risk score” OR “cox regression”}). After above filtering, 3 studies were included in this study.

### 2.7 Prognostic index construction

A prognosis index (PI) as an integrated indicator of candidate RNAs for each ccRCC patient was constructed. The PI was computed as a linear combination of the RNA expression value and weighted by LASSO Cox regression coefficients. 
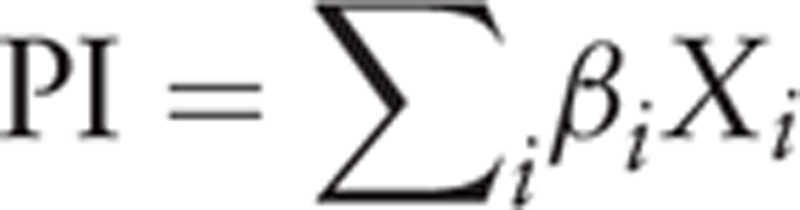


where *β*_*i*_ is the regression coefficient of the *i*th variable. *X*_*i*_ is the value of the *i*th variable. In this study, *X*_*i*_ is the log_2_-transformed expression value of each RNA and *β*_*i*_ is the LASSO Cox regression coefficient of the *i*th RNA.

### 2.8 Validation datasets construction

For assessment performance of gene signature model, gene expression array (AgilentG4502A_07_3) and RNAseq (IlluminaHiSeq) were selected. The RNAseq data were divided into 2 parts. One part includes both miRNA and mRNA samples (n = 239) that were used to be training data in this study. Another part contained all ccRCC patients (n = 510). The gene expression array contained other ccRCC patients (n = 72). All ccRCC cohorts in TCGA data were downloaded from Cancer browser (https://xenabrowser.net/datapages/) which is built by UCSC. In addition, the samples in gene expression array has little overlap in training data samples (overlap n = 5). Thus, the microarray data can be used as an independent dataset. In addition, GSE22451 is an independent dataset which downloads from GEO database.

### 2.9 Estimating performance of the models

The ability and efficiency of each model to predict ccRCC patient outcome was estimated by calculating the area under the curve (AUC) of the receiver operating characteristic (ROC), which was conducted using the survival ROC package in R software. Another indicator called concordance index (C-index) was conducted using “Hmisc” package.

### 2.10 MicroRNA target predicting

Many computational prediction approaches are available recently such as TargetScan, miRanda, PicTar, TarBase, RNAHybrid, etc, which are mostly based on complementarity, thermodynamics, or experimental validation. In this study, TargetScan (Release 7.1)^[31]^ (http://www.targetscan.org/) and miRanda (Release 19) (http://www.microrna.org/) methods were employed to predict target.^[32]^ Moreover, TargetScan and miRanda tool were used considering both conserved and nonconserved targets.

### 2.11 Gene regulation network and Gene Ontology enrichment

Each gene signature model contains a very small number of genes. It is difficult to enrich pathway through Gene Ontology (GO) analysis. Thus, the transcription factor (TF) of each gene in gene signature model is to predict and combines them to analyze to ClusterProfiler for GO analysis.^[33]^ TF target genes were identified using the approach developed by Kathrin et al,^[34]^ via defining the ±1000 bp sequence around transcription start sites as the promoter region. The genes with promoter regions completely overlapped with TF binding sites were considered as TF targets. To further enhance the reliability of TF, we calculated the correlation between predicted TF and target genes in TCGA dataset. Pearson correlation was used to estimate the relation between TFs and targets. Generally, TFs were considered to promote the expression of their targets. So, TFs expressions have positive correlations with their target genes (*r* > 0.3), and visualization of regulation network is used by Cytoscape software (version 3.5.1).^[35]^

## 3 Result

### 3.1 Demographic and clinical factors

In this study, 4 gene signature models were validated in 4 datasets, and baseline information of patients with ccRCC is listed in Table 1. The cohorts include 329 samples that both including miRNAs and mRNAs expression is used to train prognostic model. Others were employed to testing datasets. Of these datasets, TCGA with Agilent G450 platform was considered as an independent dataset which has little intersection from TCGA with Illumina Hiseq platform. For identification RNAs that significantly associated with overall survival (OS), the clinical factors of training dataset (329 samples) were analyzed. Eight clinical factors were assayed by univariate survival analysis (the 2-sided log-rank test), including age at initial diagnosis, sex, grade, adjuvant treatment, laterality, T stage, Topography, Lymph Node and Metastasis (TNM) stage, and tumor status. The results of log-rank test showed that age, grade, and T stage were significantly associated with OS in ccRCC. Multivariate Cox regression analysis of these factors suggested that grade and T stage were independent factors correlated with OS (Table 2).

**Table 1:**
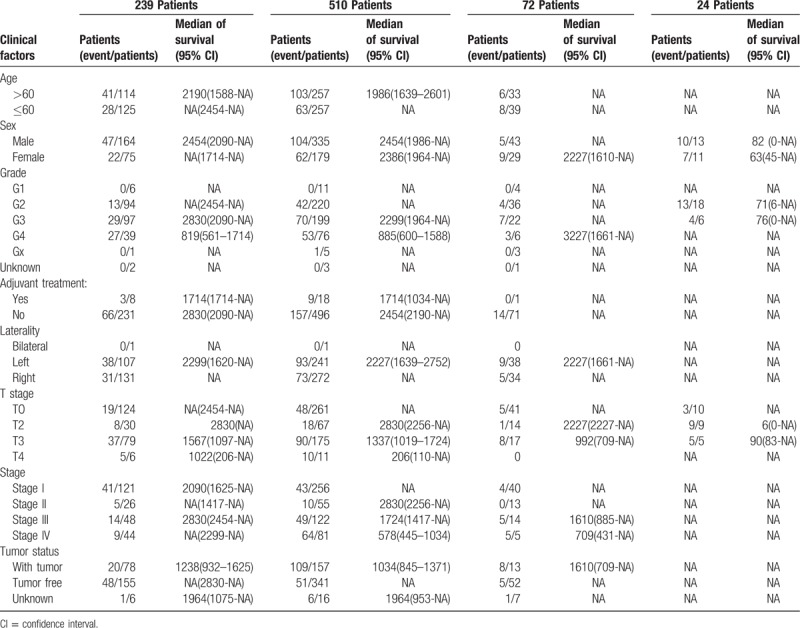
Clinical factor information of 4 clear cell renal cell carcinoma cohort datasets.

**Table 2:**
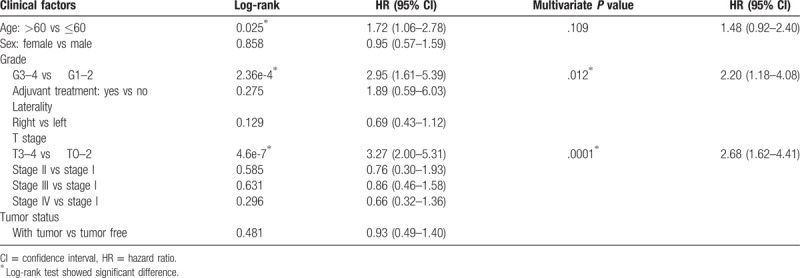
Clinical factor log-rank and multivariate Cox regression test.

### 3.2 Integrative miRNA and mRNA model in TCGA ccRCC

With LASSO combined multivariate Cox regression, 6 RNAs were obtained, and the result is listed in Table 3. Of these 6 RNAs, 3 mRNA and 1 miRNA were protective RNAS (HRs < 1) and the other 2 mRNAs were risky RNAs (HRs > 1), and the coefficient of multivariate Cox regression is applied to calculate PI for ccRCC.

**Table 3:**
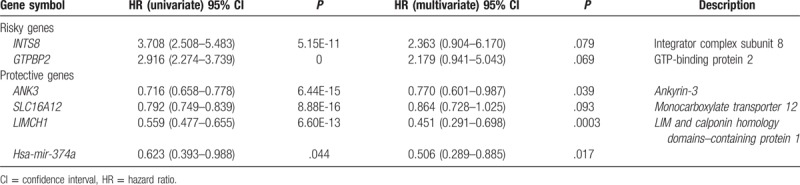
List of high- and low-risk candidate genes by Cox regression analysis with clear cell renal cell carcinoma (n = 239).

**Figure 1:**
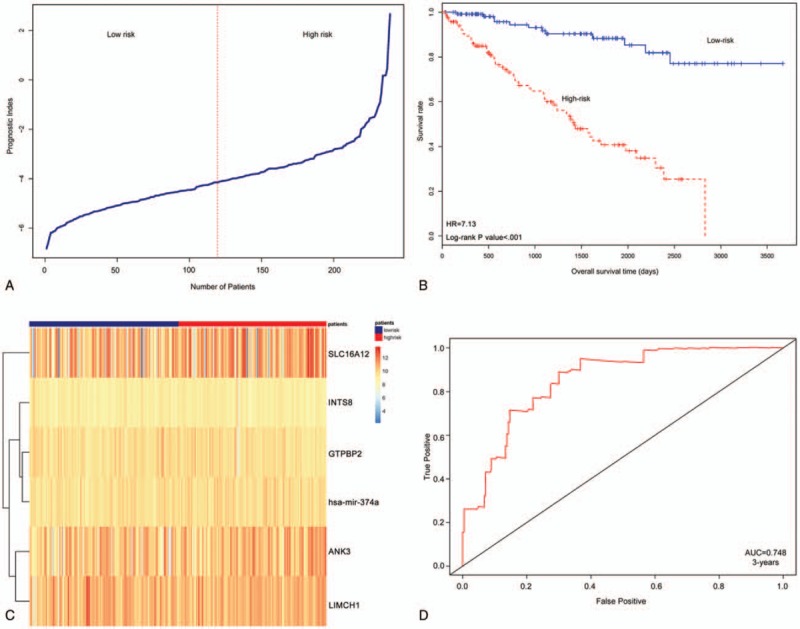
The integrative model for predicting outcome of clear cell renal cell carcinoma (ccRCC) in the cancer genome atlas (TCGA) cohort. A, Median of prognosis index (PI) value is as a sign for classification ccRCC patients into low-risk and high-risk groups. B, The heatmap of 6 RNAs in 329 patients. C, Survival analysis of low-risk and high-risk groups. D, Receiver operating characteristic (ROC) curve for estimating the effect of PI for classification of patients. CI = confidence interval, HR =hazard ratio.

As a linear combination of the expression values of 6 RNAs, the PI was significantly associated with OS in ccRCC [HR = 7.13, 95% confidence interval (CI) = 3.71–13.70, *P* < .001]. The HR of PI was greater than HRs of grade (HR = 2.20, 95% CI = 1.18–4.08, *P* = .012) or T stage (HR = 2.68, 95% CI = 1.62–4.41, *P* < .001). The patients with ccRCC were ranked by PI value (Fig. 1A). The median of PI value as threshold can classify patients into high-risk group and low-risk group. The result showed that the gene signature can significantly classify survival time of ccRCC patients (Fig. 1B). The survival time of high-risk group is significant shorter than low-risk by log-rank test (*P* < .001). The identified RNA expressions in high-risk and low-risk are listed in Figure 1C, and the value of AUC = 0.748 (3 years) demonstrated that the model performed well in predicting prognosis of ccRCC (Fig. 1D).

### 3.3 Validating the result in independent dataset

**Figure 2:**
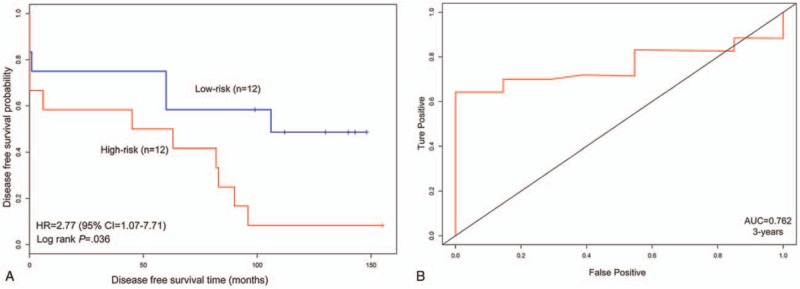
The integrative model for predicting outcome of clear cell renal cell carcinoma (ccRCC) in gene expression omnibus (GEO) cohort. A, Survival analysis of low-risk and high-risk groups in GEO dataset. B, Receiver operating characteristic (ROC) curve for estimating the effect of prognosis index (PI) for classification of patients. CI = confidence interval, HR =hazard ratio.

For validation of the result, the GSE22541 dataset is employed to be an independent data to test above result. The dataset contains 24 primary ccRCC tumor and disease-free survival (DFS) time of patients. Although this data set does not have OS time, DFS data can also reflect patient outcomes. We just employed mRNA data to validate the results due to lack of microRNA data. The validation result is shown in Figure 2.

From Figure 2, we find that 5 mRNAs can significantly classify 2 groups into high-risk and low-risk (*P* = .03). The PI was significantly associated with DFS in independent data of ccRCC (HR = 2.77, 95% CI = 1.07–7.71). The value of AUC = 0.762 (3 years) also indicated that the model performed well. Above results demonstrated that the integrative model could effectively classify patients.

### 3.4 Other gene signature of ccRCC performance in 4 datasets

For further validation the result, we tested the model in other ccRCC data in TCGA. Moreover, we also validate the other 3 models in 4 data sets. These 3 gene signature models that were published previously are listed in Table 4.

**Table 4:**
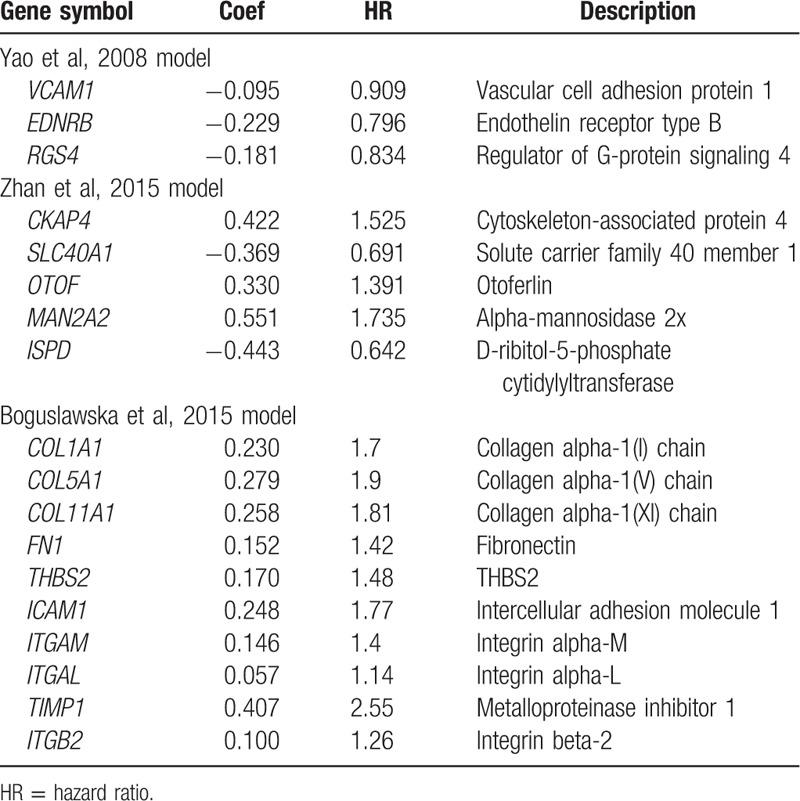
The published 3 prognostic gene signature models of clear cell renal cell carcinoma.

**Figure 3:**
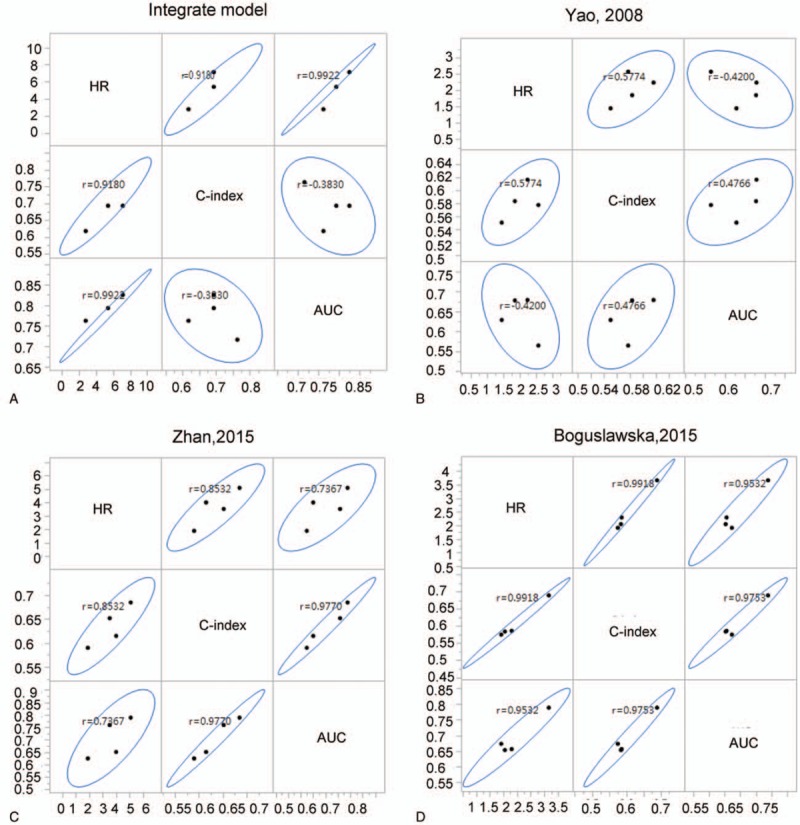
correlation analysis of 3 indicators. A, The correlation of 3 indicators in integrated model. B, The correlation of three indicators in Yao model. C, The correlation of 3 indicators in Zhan model. D, The correlation of 3 indicators in Boguslawska model. AUC = area under the curve, HR = hazard ratio.

For estimating the performance of various gene signature models, 3 indicators (HR, C-index, and AUC) of prognostic models need to be calculated. These 3 indicators were analyzed correlation in each other. Thus, the relationship of these 3 indicators in 4 models was assayed (Fig. 3).

The 3 indicators of Boguslawska model showed the strongest collinearity (Fig. 3D). The collinearity represented the model has good generalization ability. In addition, 1 value of HR in our study is missing. The integrate model from our study showed null value in TCGA_GA450 dataset. Because low-risk group that classified by integrate model has no end event occurs.

**Figure 4:**
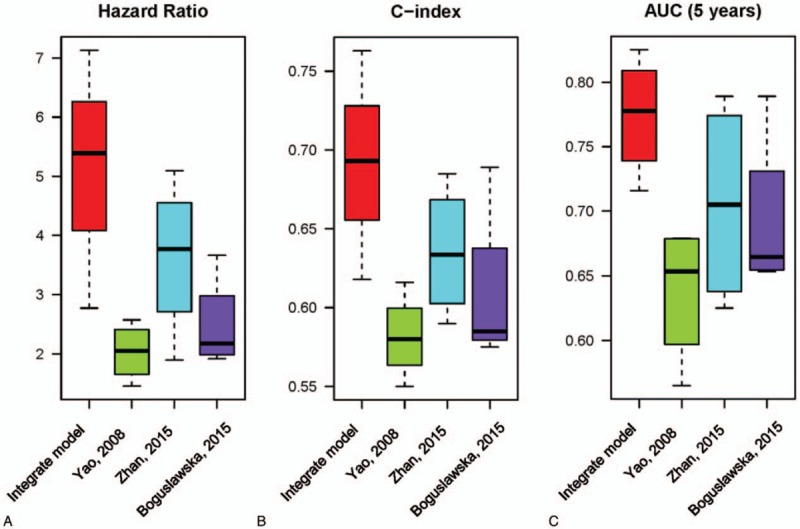
Box plot of 3 indicators [hazard ratio (HR), C-index, AUC) in different datasets. A, Box plot of HRs from 4 models distribute in different datasets. B, Box plot of C-indexes from 4 models distribute in different datasets. C, Box plot of AUCs from 4 models distribute in different datasets. AUC = area under the curve.

For testing performance of gene signature models, the box plot was employed to test the variation among indicators. Therefore, we consider 3 indicators (HR, C-index, and AUC) to evaluate the effect of all models in the 4 datasets (Fig. 4). These 3 indicators usually indicate the capacity of model prediction and high level of these indicators represents better performance of the model. The box plot also indicated the dispersion of gene signature models in different data sets. The results showed that the integrate model from our work had higher HR, C-index, and AUC among all datasets.

### 3.5 Gene Ontology enrichment of 4 gene signature models

**Figure 5:**
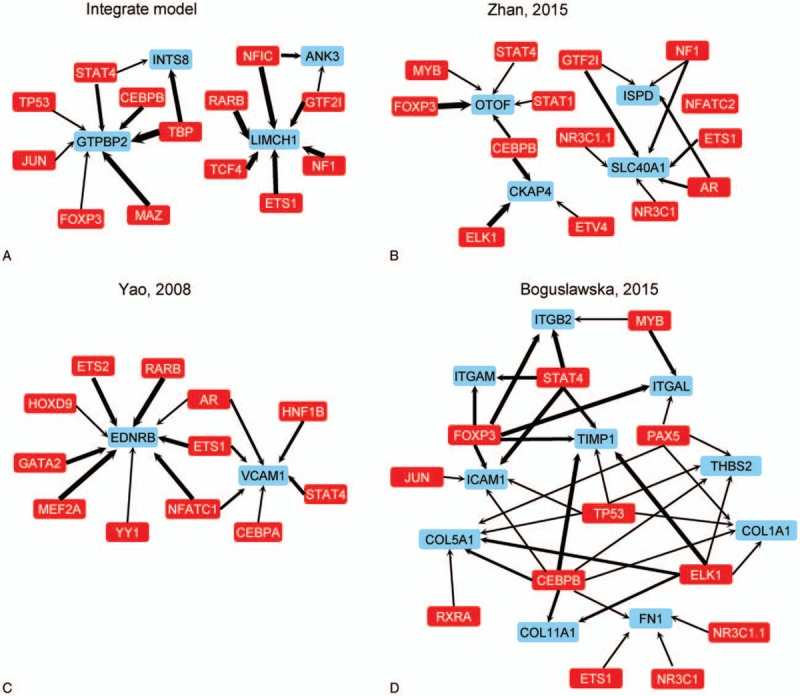
The gene regulation network of 4 gene signature models. The red blocks represent transcription factor (TF) and blue blocks represent target (genes in each gene signature). A, The gene regulation network and pathway analysis of integrate model in the cancer genome atlas (TCGA) dataset. B, The gene regulation network and pathway analysis of Zhan et al model in TCGA dataset. C, The gene regulation network and pathway analysis of Yao et al model in TCGA dataset. D, The gene regulation network and pathway analysis of Boguslawska et al model in TCGA dataset.

The results in Figure 4 show that 3 indicators of the integrated model were higher than those of other models. Thus, we try to analyze the GO enrichment and pathways in which these models involved in. Of these gene signature models, the number of genes in a gene signature model is so small that it is difficult to enrich in GO analysis. Therefore, TF of these genes in gene signature was involved in pathway analysis. The regulation network of TF and genes was constructed by method section (Fig. 5). The integrate gene signature model from our work showed that 4 genes were regulated by 13 TFs (Fig. 5A). The width of lines represented the weighted of regulation by correlation coefficient of their expression level. Regulation network of other gene signature models are listed in Figure 5B, C, and D, respectively. The results showed that these genes shared some common TFs such as STAT4, ETS1, and FOXP3.

For further investigating the GO enrichment and pathway of these genes and TFs, ClusterProfiler package was employed to analyze 4 models. The above package can compare the results of biological process, cellular component, molecular function, and KEGG pathway in 4 models (Fig. 6  ). The results of biological process suggested that the 4 gene signature models share many similar processes (Fig. 6  A). The molecular function of these gene signature models showed that integrate model was similar to model of Boguslawska. And model of Zhan was similar to model of Yao (Fig. 6  B). The molecular function enrichment showed that 4 models were very similar (Fig. 6  C). In KEGG pathway, the comparison results showed that the integrative model is involved in more cancer-associated pathways (Fig. 6  D). The model of Boguslawska et al showed very complex and mainly involved in many signaling pathways associated with cancer. Although these gene signature models and TFs are very different, the biological process and pathways were very similar.

**Figure 6:**
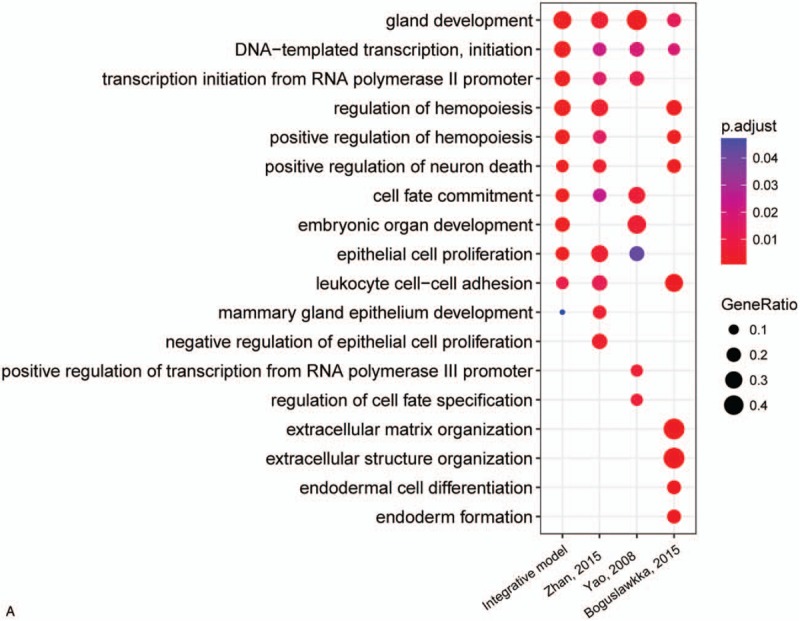
Gene Ontology (GO) and KEGG pathway enrichment comparison in 4 models. A, Biological process enrichment comparison in 4 models. B, Cellular component enrichment comparison in 4 models. C, Molecular function enrichment comparison in 4 models. D, KEGG pathways enrichment comparison in 4 models.

**Figure 6 (Continued):**
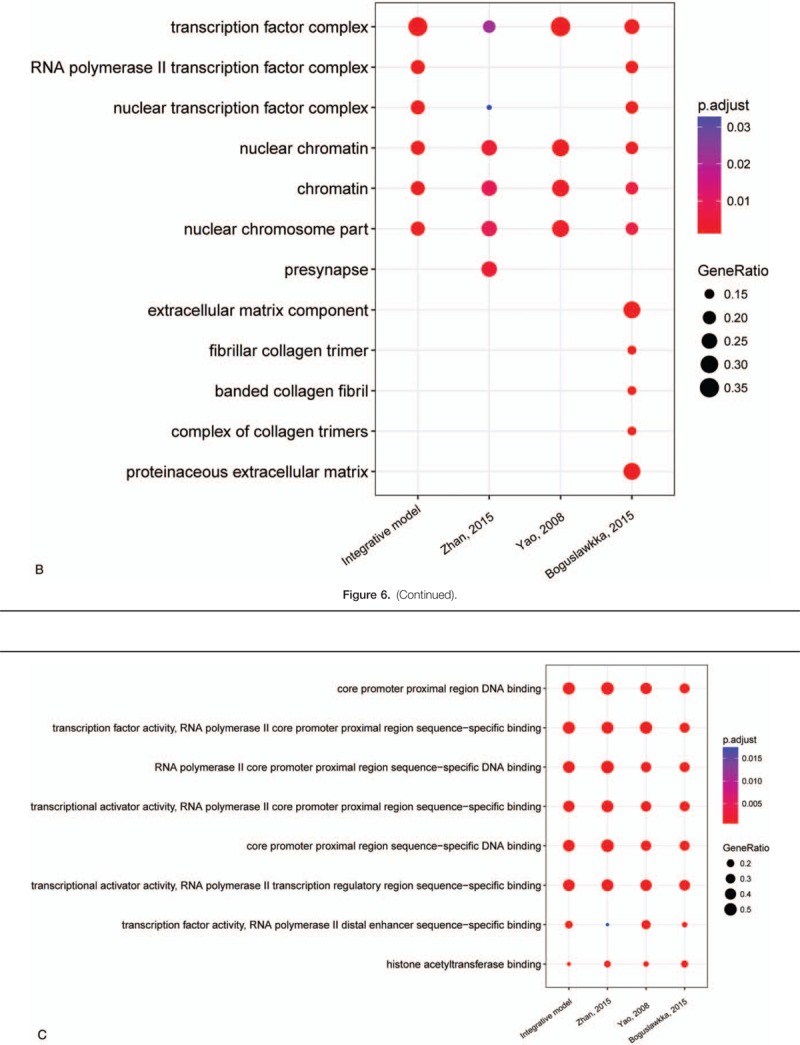
Gene Ontology (GO) and KEGG pathway enrichment comparison in 4 models. A, Biological process enrichment comparison in 4 models. B, Cellular component enrichment comparison in 4 models. C, Molecular function enrichment comparison in 4 models. D, KEGG pathways enrichment comparison in 4 models.

**Figure 6 (Continued):**
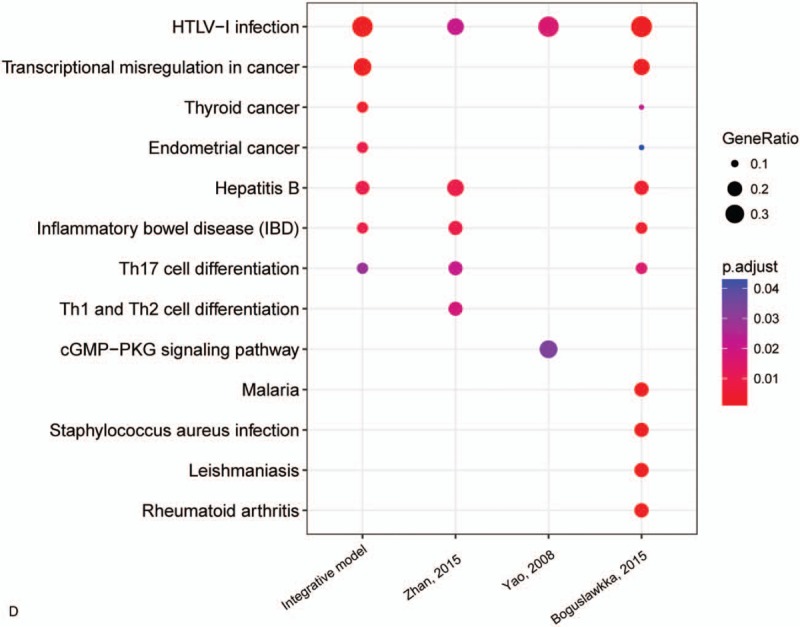
Gene Ontology (GO) and KEGG pathway enrichment comparison in 4 models. A, Biological process enrichment comparison in 4 models. B, Cellular component enrichment comparison in 4 models. C, Molecular function enrichment comparison in 4 models. D, KEGG pathways enrichment comparison in 4 models.

## 4 Discussion

Our present study combined LASSO and multivariate Cox regression to calculate a prognostic gene signature model from integrative microRNA and mRNA expression of TCGA dataset. The other platform of TCGA and GEO dataset as validation datasets were employed to validate the results. Previous study has provided many biomarkers for predicting prognosis of ccRCC. In this study, we proposed 5 mRNAs and 1 microRNA (*INTS8*, *GTPBP2*, *ANK3*, *SLC16A12*, *LIMCH1*, and *hsa-mir-374a*) as robust gene signature model that could effectively predict the prognosis for ccRCC. In addition, we also found a regulation pair of hsa-mir-374a and *ANK3* from TargetScan.

Of these genes, *INTS8*, *ANK3*, and *LIMCH1* indicated that they are associated with renal cancer by previous publication.^[36–38]^ To the best of our knowledge, we did not find the *GTPBP2* and *SLC16A12* associated with kidney cancer. Although the gene *hsa-mir-374a* is associated with cancer in many reports, there is no study on ccRCC. Previous studies have shown that hsa-mir-374a (HR = 0.64, 95% CI: 0.48–0.86) can reduce the risk of colorectal cancer.^[39]^ Our findings in kidney cancer also showed similar results (HR = 0.51, 95% CI: 0.29–0.89), so we hypothesized that hsa-mir-374a could reduce the risk of death. These 6-gene signatures showed robust ability in predicting prognosis of ccRCC.

Generally, gene signature prediction for prognosis mainly derived from Cox regression. However, different data preprocess and steps for Cox regression might lead to different results. This study combined the genes with differential expression, univariate Cox regression, LASSO, and multivariate Cox regression method to obtain gene signature for prognosis of ccRCC. In addition, 3 indictors including HR, C-index, and value of AUC were employed to estimate all models in systems level (Fig. 5). The results showed that the integrate model had more advantages than others.

Although our results show more advantages, it does not mean that other models are not good. Among the various gene signature models previously proposed, prognosis is thought to be predictive. In fact, different gene signature has similar pathway and its own special function. The similar pathways are possible to perform similar functions that affect prognosis. The different pathways may represent the heterogeneity of ccRCC.

In the work, the integrate model mainly involved in viral infection and inflammatory bowel disease (IBD)-related pathways. From literature review, there are few reports about viral infection associated with ccRCC. However, there are many reports about the relationship between IBD and renal cancer^[40,41]^. Although this work could not reveal the relationship between IBD and prognosis of ccRCC, the result might provide a new insight for further study about the ccRCC.

In addition, the gene expression data and clinical data of available ccRCC are very limited, which results in difficulty to further verify. We just used different platforms of TCGA dataset and GEO dataset as independent datasets for training and validation. For further validation of different, we test other 3 gene signature models (from Cox regression method) in different datasets. Moreover, the integrate model indeed showed greater stability and versatility in the TCGA and GSE22541 datasets.

Despite the limited data available, the data we obtained may have bias. However, the gene markers obtained by LASSO coupled multivariate Cox regression are indeed more stable in various public databases. In this study, we propose the optimization steps for analyzing gene prognostic markers by Cox regression. In addition, when gene markers are too scarce to enrich their functions by GO analysis, we can further analyze GO functional enrichment by predicting their TFs. We expect to find more and more stable genetic markers by this way to provide a more scientific reference for drug development and clinical decision-making.

## Acknowledgment

The publications retrieval from staff in Evidence Based Medicine Center is appreciated by the authors.

## Author contributions

**Conceptualization:** Peng Chang, Kehu Yang.

**Data curation:** Jingyun Zhang.

**Formal analysis:** Peng Chang, Juan Ling.

**Funding acquisition:** Zhitong Bing.

**Investigation:** Jinhui Tian, Xiuxia Li, Yumin Li.

**Methodology:** Peng Chang, Xiuxia Li.

**Project administration:** Juan Ling.

**Resources:** Jingyun Zhang, Long Ge.

**Software:** Zhitong Bing, Jinhui Tian, Jingyun Zhang, Long Ge.

**Supervision:** Kehu Yang.

**Validation:** Zhitong Bing.

**Visualization:** Jinhui Tian, Yumin Li.

**Writing – original draft:** Peng Chang, Zhitong Bing, Yumin Li.

**Writing – review and editing:** Peng Chang, Kehu Yang.
